# The Advisory Committee on Immunization Practices’ Interim Recommendation for Use of Pfizer-BioNTech COVID-19 Vaccine in Children Aged 5–11 Years — United States, November 2021

**DOI:** 10.15585/mmwr.mm7045e1

**Published:** 2021-11-12

**Authors:** Kate R. Woodworth, Danielle Moulia, Jennifer P. Collins, Stephen C. Hadler, Jefferson M. Jones, Sujan C. Reddy, Mary Chamberland, Doug Campos-Outcalt, Rebecca L. Morgan, Oliver Brooks, H. Keipp Talbot, Grace M. Lee, Beth P. Bell, Matthew F. Daley, Sarah Mbaeyi, Kathleen Dooling, Sara E. Oliver

**Affiliations:** ^1^CDC COVID-19 Response Team; ^2^Tanaq Support Services, LLC, St. George Tanaq Corporation, Anchorage, Alaska; ^3^University of Arizona, College of Medicine, Phoenix, Arizona; ^4^Department of Health Research Methods, Evidence and Impact, Hamilton, Ontario, Canada; ^5^Watts Healthcare Corporation, Los Angeles, California; ^6^Vanderbilt University School of Medicine, Nashville, Tennessee; ^7^Stanford University School of Medicine, Stanford, California; ^8^University of Washington, Seattle, Washington; ^9^Institute for Health Research, Kaiser Permanente Colorado, Denver, Colorado.

The Pfizer-BioNTech COVID-19 (BNT162b2) vaccine is a lipid nanoparticle–formulated, nucleoside-modified mRNA vaccine encoding the prefusion spike glycoprotein of SARS-CoV-2, the virus that causes COVID-19. On August 23, 2021, the Food and Drug Administration (FDA) approved a Biologics License Application (BLA) for use of the Pfizer-BioNTech COVID-19 vaccine, marketed as Comirnaty (Pfizer, Inc.), in persons aged ≥16 years (*1*). The Pfizer-BioNTech COVID-19 vaccine is also recommended for adolescents aged 12–15 years under an Emergency Use Authorization (EUA) (*1*). All persons aged ≥12 years are recommended to receive 2 doses (30 *μ*g, 0.3 mL each), administered 3 weeks apart (*2*,*3*). As of November 2, 2021, approximately 248 million doses of the Pfizer-BioNTech COVID-19 vaccine had been administered to persons aged ≥12 years in the United States.* On October 29, 2021, FDA issued an EUA amendment for a new formulation of Pfizer-BioNTech COVID-19 vaccine for use in children aged 5–11 years, administered as 2 doses (10 *μ*g, 0.2 mL each), 3 weeks apart (Table) (*1*). On November 2, 2021, the Advisory Committee on Immunization Practices (ACIP) issued an interim recommendation^†^ for use of the Pfizer-BioNTech COVID-19 vaccine in children aged 5–11 years for the prevention of COVID-19. To guide its deliberations regarding recommendations for the vaccine, ACIP used the Evidence to Recommendation (EtR) Framework^§^ and incorporated a Grading of Recommendations, Assessment, Development and Evaluation (GRADE) approach.^¶^ The ACIP recommendation for the use of the Pfizer-BioNTech COVID-19 vaccine in children aged 5–11 years under an EUA is interim and will be updated as additional information becomes available. The Pfizer-BioNTech COVID-19 vaccine has high efficacy (>90%) against COVID-19 in children aged 5–11 years, and ACIP determined benefits outweigh risks for vaccination. Vaccination is important to protect children against COVID-19 and reduce community transmission of SARS-CoV-2.

**TABLE:** COVID-19 vaccines approved or authorized by the Food and Drug Administration for persons aged <18 years — United States, November 2021*

Age group at vaccination, yrs	Vaccine manufacturer	Vial cap color	Concentration of mRNA per dose	Injection volume	Diluent^†^ volume	Doses per vial
5–11	Pfizer-BioNTech	Orange	10 *μ*g	0.2 mL	1.3 mL	10
12–17	Pfizer-BioNTech	Purple	30 *μ*g	0.3 mL	1.8 mL	6

* Both Pfizer-BioNTech COVID-19 vaccines are administered intramuscularly as 2 doses with a recommended interval of 21 days between doses. Additional information regarding each Pfizer-BioNTech formulation (e.g., ingredients and storage conditions) as well as educational materials and information regarding other Food and Drug Administration–approved or -authorized COVID-19 vaccines is available at https://www.cdc.gov/vaccines/covid-19/clinical-considerations/covid-19-vaccines-us.html.

^†^ Diluent for both formulations is 0.9% sterile sodium chloride injection, USP (nonbacteriostatic).

Since June 2020, ACIP has convened 21 public meetings to review data relevant to the potential use of COVID-19 vaccines, including the Pfizer-BioNTech COVID-19 vaccine.** In addition, the ACIP COVID-19 Vaccines Work Group, comprising experts in infectious diseases, vaccinology, vaccine safety, public health, and ethics, has held weekly meetings to review COVID-19 surveillance data, evidence for vaccine efficacy and effectiveness, safety, and implementation considerations for COVID-19 vaccines. Within the EtR Framework for the Pfizer-BioNTech COVID-19 vaccine for children aged 5–11 years, ACIP considered the importance of COVID-19 as a public health problem, as well as benefits and harms, parents’ values and preferences, acceptability, feasibility, resource use, and equity for use of the vaccine among children. After conducting a systematic review of published and unpublished evidence for benefits and harms, the Work Group used the GRADE approach to assess the certainty of evidence for outcomes related to the vaccine, rated on a scale of type 1 (high certainty) to type 4 (very low certainty).^††^ Work Group conclusions regarding evidence for the Pfizer-BioNTech COVID-19 vaccine were presented to ACIP at a public meeting on November 2, 2021.

The body of evidence regarding immunogenicity, efficacy, and safety of the Pfizer-BioNTech COVID-19 vaccine among children aged 5–11 years was primarily composed of data from one randomized, double-blind, placebo-controlled phase II/III clinical trial that initially enrolled 2,268 participants aged 5–11 years, randomized 2:1 to receive vaccine or saline placebo (*1*). Interim findings from this clinical trial were based on data from participants with a median follow-up of 3.3 months. Vaccine efficacy was supported by two types of evidence: direct efficacy against symptomatic infection and immunobridging data consisting of neutralizing antibody titers from vaccine recipients aged 5–11 years who received 2 doses of 10 *μ*g each compared with those from vaccine recipients aged 16–25 years who received 2 doses of 30 *μ*g each. Vaccine efficacy was 90.9% (95% CI = 68.3%–98.3%) in preventing symptomatic, laboratory-confirmed COVID-19 in children aged 5–11 years with or without evidence of previous SARS-CoV-2 infection, based on infection in three vaccine recipients and 16 placebo recipients, none of whom were hospitalized. The measure of immune response to 2 doses of the Pfizer-BioNTech COVID-19 vaccine in children aged 5–11 years without evidence of previous SARS-CoV-2 infection was at least as high as the response observed in persons aged 16–25 years, with a geometric mean ratio for 50% neutralizing antibody titer of 1.04 (95% CI = 0.93–1.18), satisfying the noninferiority criteria.^§§^ Among vaccine recipients aged 5–11 years, reactogenicity symptoms, defined as solicited local injection site or systemic reactions during the 7 days after vaccination, were frequent (86.2% of vaccine recipients reported any local reaction, and 66.6% reported any systemic reaction); the vast majority were mild to moderate. Reactogenicity symptoms were generally less frequent in children aged 5–11 years than in persons aged 16–25 years. Systemic adverse reactions were more commonly reported after the second dose than after the first dose, had a median onset of 1–2 days after vaccination, and resolved in a median of 1–2 days. Severe local and systemic adverse reactions (grade 3 or higher, defined as interfering with daily activity) occurred in 2.7% of vaccine recipients and 1.1% of placebo recipients. Among vaccine recipients who repor

ted any reaction of grade 3 or higher, the most common symptoms were fatigue (0.9%), headache (0.3%), fever (0.8%) and injection site pain (0.6%). Overall, reactions of grade 3 or higher were also more commonly reported after the second dose than after the first dose. The prevalence of related adverse events was lower in children who were seropositive at baseline (two of 133; 1.5%) compared with the prevalence in those who were seronegative at baseline (44 of 1,385; 3.2%); in addition, individual local and systemic reactions were less common in seropositive children. Serious adverse events^¶¶^ were uncommon and occurred with similar frequency among vaccine (0.07%) and placebo (0.10%) recipients, with no statistically significant difference in frequency observed between the two groups. An expanded safety cohort of 2,379 children (including 1,591 vaccine recipients) was added to monitor for serious adverse events, which had a median follow-up of 2.4 weeks after receipt of the second dose. No serious adverse events related to the vaccination were identified in either group, and no specific safety concerns were identified among vaccine recipients aged 5–11 years. A detailed summary of safety data, including information on reactogenicity, is available at https://www.cdc.gov/vaccines/covid-19/info-by-product/pfizer/reactogenicity.html.

From the GRADE evidence assessment, the level of certainty for the benefits of Pfizer-BioNTech COVID-19 vaccination among children aged 5–11 years was type 1 (high certainty) for the prevention of symptomatic laboratory-confirmed COVID-19. Regarding potential harms after vaccination, evidence was type 4 (very low certainty) for serious adverse events because of small sample size and short follow-up time and type 2 (moderate certainty) for reactogenicity for imprecision. No data were available to assess the other GRADE benefits, specifically prevention of hospitalization for COVID-19, prevention of multisystem inflammatory syndrome in children (MIS-C), or prevention of asymptomatic SARS-CoV-2 infection.

Data reviewed within the EtR Framework supported the use of the Pfizer-BioNTech COVID-19 vaccine in children aged 5–11 years. ACIP concluded that COVID-19 in children is a major public health problem. Approximately 1.9 million COVID-19 cases and 8,300 hospitalizations among U.S. children aged 5–11 years had been reported to CDC as of October 10, 2021 (*5*). As of October 4, 2021, CDC had received reports of 5,217 cases of MIS-C, a severe hyperinflammatory syndrome occurring several weeks after acute SARS-CoV-2 infection; 44% of MIS-C cases have occurred in children aged 5–11 years.*** In addition, children aged 5–11 years represent a growing proportion of new COVID-19 cases reported to CDC, accounting for 10.6% of infections for the week of October 10, 2021, although children aged 5–11 years represent 8.7% of the population (*4*). In addition, children can contribute to transmission of SARS-CoV-2 in households and communities (*5*,^6^). A study of residual sera from commercial laboratories in 47 U.S. jurisdictions estimated the seroprevalance in this age group to be 38% as of September 2021 (*7*). As of October 14, 2021, the cumulative COVID-19–associated hospitalization rate for children aged 5–11 years over the course of the pandemic was 28.6 per 100,000 population,^†††^ which is similar to the influenza-associated hospitalization rate for the same age group during the 2017–18, 2018–19, and 2019–20 influenza seasons (24.3–31.7 per 100,000 population), despite intensive mitigation efforts in place during the COVID-19 pandemic not present during previous influenza seasons.^§§§^ During January 1, 2020–October 16, 2021, 94 COVID-19–associated deaths among children aged 5–11 years were reported to CDC’s National Center for Health Statistics, representing 1.7% of all deaths in this age group during the same period; COVID-19 ranks as the eighth leading cause of death in this age group (*8**,**9*). Post-COVID conditions, a range of new, worsening, or ongoing health problems after SARS-CoV-2 infection, have been reported in children (*10*). During the 2020–21 school year, an estimated 19,692 school closures occurred in the 50 U.S. states, affecting approximately 12 million students. During August 2–October 22, 2021, approximately 2,350 schools faced COVID-19–related closures, with nearly one half resulting from COVID-19 cases among students (*11*). Several surveys suggested that 34%–57% of parents intended to have their children vaccinated (*11*). 

Implementation of this vaccine recommendation will require educating providers regarding the different formulation, dose, and volume of vaccine for use in this population to avoid vaccine administration errors. COVID-19 vaccines must be administered according to applicable state and territorial vaccination laws. ACIP determined that use of the Pfizer-BioNTech COVID-19 vaccine among children is a reasonable and efficient allocation of resources. To expand COVID-19 vaccine access, additional considerations should be given to demographic groups that have experienced disproportionate COVID-19 morbidity and mortality, as well as those with barriers to routine health care (e.g., members of certain racial/ethnic groups and those living in a rural or frontier area, experiencing homelessness, with a disability, or lacking health insurance). Children from racial and ethnic minority groups have experienced a disproportionally high incidence of COVID-19 as well as secondary impacts of the COVID-19 pandemic such as reduced in-person learning (*12*). Providing rapid and equitable access to COVID-19 vaccines for children will necessitate increasing the enrollment of pediatric health care providers into the COVID-19 vaccination program, using the broad geographic accessibility of pharmacies, and expanding school-focused strategies to ensure vaccination opportunities for a diverse population, as well as engagement with community leaders, pediatric health care providers, and parents or guardians.

The GRADE evidence profile, which provides details on the identification and assessment of relevant evidence, and EtR-supporting evidence are available at https://www.cdc.gov/vaccines/acip/recs/grade/covid-19-pfizer-age-5-11-eua.html and https://www.cdc.gov/vaccines/acip/recs/grade/covid-19-pfizer-age-5-11-eua-etr.html. Additional clinical considerations are available at https://www.cdc.gov/vaccines/covid-19/info-by-manufacturer/pfizer/clinical-considerations.html.

ACIP reviewed the balance of benefits and risks regarding vaccination of children aged 5–11 years, considering evidence around both known and potential benefits and risks. Myocarditis is a rare adverse event that has been reported after receipt of mRNA COVID-19 vaccines (*13*). The observed risk is highest in males aged 12–29 years.^¶¶¶^ No cases of myocarditis were reported among 3,082 trial participants aged 5–11 years with ≥7 days of follow-up after receipt of dose 2, although the study was not powered to assess the risk for myocarditis (*1*). The baseline (before the COVID-19 pandemic) risk for myocarditis is much higher in adolescents aged 12–17 years than in children aged 5–11 years.**** Therefore, myocarditis after receipt of an mRNA COVID-19 vaccine by adolescents might not predict risk for myocarditis in younger children. Regardless of seropositivity rates, ACIP determined that the benefits of COVID-19 vaccination outweigh the known and potential risks. Vaccination after infection significantly enhances protection and further reduces risk for reinfection; ^††††^ no concerns have been identified in postauthorization safety surveillance associated with vaccination of seropositive persons aged ≥12 years. Children can experience significant morbidity, such as MIS-C and post-COVID sequelae, after mild or asymptomatic infection (*7*). Further, Delta-wave surges of pediatric COVID-19 hospitalizations occurred even with a significant proportion of children who were seropositive at that time (*7*). After assessing the balance of benefits and risks for COVID-19 vaccination in children aged 5–11 years, ACIP made an interim recommendation for vaccination in this population as authorized under the EUA.

The interim recommendation and clinical considerations are based on use of the Pfizer-BioNTech COVID-19 vaccine under an EUA and might change as more evidence becomes available. Before vaccination, the EUA Fact Sheet should be provided to parents or guardians. ACIP will continue to review additional data as they become available; updates to recommendations or clinical considerations will be posted on the ACIP website (https://www.cdc.gov/vaccines/hcp/acip-recs/vacc-specific/covid-19.html).

## Reporting of Vaccine Adverse Events

FDA requires that vaccination providers report vaccination administration errors, serious adverse events, cases of multisystem inflammatory syndrome, and cases of COVID-19 that result in hospitalization or death after administration of COVID-19 vaccine under an EUA (*1*). Adverse events that occur after receipt of any COVID-19 vaccine should be reported to the Vaccine Adverse Event Reporting System (VAERS). Information on how to submit a report to VAERS is available at https://vaers.hhs.gov/index.html or 1-800-822-7967. Any person who administers or receives a COVID-19 vaccine (or their parent or guardian) is encouraged to report any clinically significant adverse event, whether or not it is clear that a vaccine caused the adverse event. In addition, CDC has developed a new, voluntary smartphone-based online tool (v-safe) that uses text messaging and online surveys to provide near real-time health check-ins after receipt of a COVID-19 vaccine. Parents or guardians can register their children in v-safe and complete the health surveys on their behalf. CDC’s v-safe call center follows up on reports to v-safe that include possible medically significant health events to collect additional information for completion of a VAERS report. Information on v-safe is available at https://www.cdc.gov/vsafe.

**Summary**

**What is already known about this topic?**

On October 29, 2021, the Food and Drug Administration granted Emergency Use Authorization for the Pfizer-BioNTech COVID-19 vaccine for children aged 5–11 years.

**What is added by this report?**

On November 2, 2021, after a systematic review of available data, the Advisory Committee on Immunization Practices made an interim recommendation for use of the Pfizer-BioNTech COVID-19 vaccine in children aged 5–11 years in the United States for prevention of COVID-19.

**What are the implications for public health practice?**

The Pfizer-BioNTech COVID-19 vaccine has high efficacy (>90%) against COVID-19 in children aged 5–11 years, and benefits outweigh risks for vaccination. Vaccination is important to protect children against COVID-19 and reduce community transmission of SARS-CoV-2.

## Footnotes

* Accessed November 3, 2021. https://covid.cdc.gov/covid-data-tracker/#vaccinations

† On November 2, 2021, ACIP voted 14–0 (with one member absent) in favor of the interim recommendation for use of Pfizer-BioNTech COVID-19 vaccine for persons aged 5–11 years.

§
https://www.cdc.gov/vaccines/acip/recs/grade/downloads/acip-evidence-recs-framework.pdf

¶
https://www.cdc.gov/vaccines/acip/recs/grade/about-grade.html

**
https://www.cdc.gov/vaccines/acip/meetings/index.html

††
https://www.cdc.gov/vaccines/acip/recs/grade

§§ 1.5-fold noninferiority criterion: lower bound of the two-sided 95% CI for geometric mean ratio >0.67.

¶¶ Serious adverse events that were reported in the initial cohort of the trial included a limb fracture in one vaccine recipient and abdominal pain and pancreatitis in one placebo recipient. Serious adverse events that were reported in the expanded safety cohort included infective arthritis (infection of the knee), foreign body ingestion of a penny, and epiphyseal fracture in three children (one each) in the vaccine group.

***
https://covid.cdc.gov/covid-data-tracker/#mis-national-surveillance

†††
https://gis.cdc.gov/grasp/COVIDNet/COVID19_3.html

§§§
https://gis.cdc.gov/GRASP/Fluview/FluHospRates.html

¶¶¶
https://www.cdc.gov/vaccines/acip/meetings/downloads/slides-2021-10-20-21/07-COVID-Su-508.pdf

****
https://www.cdc.gov/vaccines/acip/meetings/downloads/slides-2021-11-2-3/04-COVID-Oster-508.pdf

††††

https://www.cdc.gov/coronavirus/2019-ncov/science/science-briefs/vaccine-induced-immunity.html
